# Implementing Deep Learning Techniques in 5G IoT Networks for 3D Indoor Positioning: DELTA (DeEp Learning-Based Co-operaTive Architecture)

**DOI:** 10.3390/s20195495

**Published:** 2020-09-25

**Authors:** Brahim El Boudani, Loizos Kanaris, Akis Kokkinis, Michalis Kyriacou, Christos Chrysoulas, Stavros Stavrou, Tasos Dagiuklas

**Affiliations:** 1 Division of Computer Science and Informatics, London South Bank University, London SE1 0AA, UK; tdagiuklas@lsbu.ac.uk; 2 Department of Electrical Engineering, Eindhoven University of Technology, 5612 AE Eindhoven, The Netherlands; l.kanaris@sigintsolutions.com (L.K.); a.kokkinis@sigintsolutions.com (A.K.); 3 Faculty of Pure and Applied Sciences, Open University of Cyprus, 2252 Nicosia, Cyprus; m.kyriacou@sigintsolutions.com (M.K.); stavros.stavrou@ouc.ac.cy (S.S.); 4 School of Computing, Edinburgh Napier University, Edinburgh EH11 4DY, UK; C.Chrysoulas@napier.ac.uk

**Keywords:** 5G IoT, indoor positioning, deep learning, tracking, localization, navigation, positioning accuracy, single access point positioning, Internet of Things

## Abstract

In the near future, the fifth-generation wireless technology is expected to be rolled out, offering low latency, high bandwidth and multiple antennas deployed in a single access point. This ecosystem will help further enhance various location-based scenarios such as assets tracking in smart factories, precise smart management of hydroponic indoor vertical farms and indoor way-finding in smart hospitals. Such a system will also integrate existing technologies like the Internet of Things (IoT), WiFi and other network infrastructures. In this respect, 5G precise indoor localization using heterogeneous IoT technologies (Zigbee, Raspberry Pi, Arduino, BLE, etc.) is a challenging research area. In this work, an experimental 5G testbed has been designed integrating C-RAN and IoT networks. This testbed is used to improve both vertical and horizontal localization (3D Localization) in a 5G IoT environment. To achieve this, we propose the DEep Learning-based co-operaTive Architecture (DELTA) machine learning model implemented on a 3D multi-layered fingerprint radiomap. The DELTA begins by estimating the 2D location. Then, the output is recursively used to predict the 3D location of a mobile station. This approach is going to benefit use cases such as 3D indoor navigation in multi-floor smart factories or in large complex buildings. Finally, we have observed that the proposed model has outperformed traditional algorithms such as Support Vector Machine (SVM) and K-Nearest Neighbor (KNN).

## 1. Introduction

In the era of 5G IoT [1], real-time positioning is becoming increasingly required by context-aware applications and location-based services. Typical scenarios include locating doctors and patients inside a hospital, advertising commercial products to mall visitors, monitoring gas and oil plants status, pinpointing dead crops in vertical farms, identifying victims’ location in Public Protection and Disaster Recovery (PPDR), etc. Moreover, several advanced applications can further provide cellular phone fraud detection, location-sensitive billing, as well as navigation from and to almost everywhere, through the utilization of heterogeneous wireless technologies, fusion of sensor and IoT data [2,3,4,5]. A recent report published by IEEE has estimated 50 billion [6] mobile devices will be connected to the cloud by the end of 2020. These devices will need constant access to data anywhere. Cisco has predicted that 26 billion [7] of these devices will be IoT or Wireless Sensor Network (WSN) devices. In this respect, technologies like Cloud Radio Access Network (C-RAN), Millimeter Wave (mm-Wave) communication, ultra dense communication [8], device-to-device (D2D) communication and Vehicle-to-everything (V2X) [9,10] and protocols like IEEE 802.11be (Extremely high Throughput WLAN) [11], IEEE 802.11az (Next Generation Positioning) [12] are not only introduced to increase the bandwidth of communication but also to offer the possibility of co-operative and precise localization. Additionally, with 5G paving the path for a seamless collaboration among heterogeneous wireless systems (cellular, WiFi, WSN, IoT, etc.), a great opportunity has risen in the area of indoor localization in urban areas under the framework of smart cities. Such high dense networks could be utilized to solve multi-agent positioning and offer agility and scalability for accurate positioning as a service. In this direction, we propose a DEep Learning-based with Co-operative Architecture (DELTA) algorithm to enhanced 3D indoor localization. The contributions of this paper can be summarized as follows:

- A realistic 3D indoor localization scenario for 5G IoT networks has been designed using an emulated 5G C-RAN and Zolertia IoT nodes.
- We present a novel approach to Received Signal Strength (RSS)-based fingerprint using 3D multi-layered radiomap to enhance the learning of network signal behaviour.
- A deep learning cooperative algorithm is implemented on the constructed multi-layered radiomap for an improved 3D localization indoor localization. The proposed method targets improving vertical and horizontal localization for use case scenarios such as indoor navigation or people tracking in multi-floor smart or large complex buildings. Based on the results of the emulated realistic radio-planning, we have shown how the DELTA outperformed KNN and SVM.

The remaining of this paper is organized as follows: Section 2 covers related research to this paper. Section 3 describes the problem related to indoor positioning in a 3D environment. Section 4 gives a detailed description of the underlying architecture of the DELTA model. Section 5 consists of a discussion and analysis of the performance results produced by our proposed approach compared with other traditional models. Lastly, Section 6 summarises a conclusion and spots possible future work.

## 2. Related Work

Indoor positioning techniques can be divided into two main categories: fingerprint and multilateration. In the latter, given a known propagation speed, the distance between a receiver and a group of transmitters is measured using techniques such as Direction of Arrival (DoA), Time of Arrival (TOA)/Time of Flight (TOF), Angle of Arrival (AoA), Time Difference of Arrival (TDOA) and Return Time of Flight (RTOF). These techniques are commonly used in Global Navigation Satellite Systems (GNSS) [13], such as Global Positioning System (GPS) and Galileo, but surprisingly they are also found in IoT indoor navigation solutions [14]. However, multilateration relies mainly on the travelling time or the direction of the signal rays. This makes indoor localization a complex task especially with many issues rising such as synchronization errors and multi-path fading [15,16,17].

In the fingerprint-based technique, a set of RSS measurements are taken and linked to specific Reference Points (RP) (also known as fingerprints or signatures). Localization using this approach works in two phases: offline and online. During the offline phase, a site survey is conducted with the purpose of linking the measured signal strength values to predefined RPs. The outcome of this measurements campaign is then stored in a radiomap database. During the online phase, a user equipment receives real-time signals and tries to match them with existing records stored in the radiomap database using a matching algorithm. In the context of IoT localization, the RSS signal is collected from wireless technologies such as Zigbee, LoRA, Wifi, Raspberry Pi, BLE, RFID. Since it does not require any specialised equipment or time synchronization to obtain the RSS signal, this technique is usually preferred to multilateration. For instance, authors in [18] have studied how robust localization for robots and IoT can be achieved using RSS fingerprint. Additionally, another interesting approach has been introduced in [19] where the authors have focused on the use of IoT and Wifi-enabled devices to improve fingerprinting in an indoor environment. Recently, a new concept has been developed by Ali et al. [20] using raster maps instead of traditional offline scene analysis. Furthermore, a hybrid solution implemented on LoRa devices, which combines RSS fingerprinting with AoA methods is discussed in [14]. The proposed idea is very promising but it has inherited synchronization issues from multilateration. From these examples, it is undoubtedly clear that the RSS-based fingerprint method is widely used in the research community. This is due to improved localization and reduced computational complexity, as concluded by Amr et al. [19]. A detailed comparison of technologies and algorithms implementing the fingerprint technique for IoT indoor positioning has been carried out by [15,21,22,23].

In the fingerprint-based approach, deep learning techniques have been widely used to extract common patterns from a sparse radiomap database and to improve localization. In recent years, it has gained a huge popularity among the indoor localization researchers, in particular, due to its robustness and high accuracy [24]. Supervised and unsupervised deep learning algorithms have been recently implemented in 2D localization [25] and multi-floor localization [26]. Recently, Wafa et al. [27] studied the use of Convolutional Neural Networks (CNN) on IoT-Sensor System to determine the node location. In this simulation, the authors converted the 2D localization problem into a 3D image tensor identification problem. The 3D tensor has been constructed using a 2D matrix of RSS signals and 1D kurtosis. This concept has achieved 2 m average error accuracy but a similar system was also implemented in [28] and usually requires a large number of access points deployed in a small space to achieve this result. In [29], authors have implemented a Deep Belief Network (DBN) on an active RFID tag system for accurate location estimation. Their solutions consisted of a set of stacked Restricted Boltzmann Machine (RBM) layers called autoencoders trained using Contrastive Divergence with one-step iteration (CD-1). This algorithm has improved the 2D positioning. To achieve this, the authors have deployed a large number of RFID tags in a 12 m × 12 m indoor environment, which does not take into account the power consumption of the devices. Finally, Wang et al. [30] have suggested a hybrid deep learning solution combining a regression Deep Neural Network (DNN) with a Convolutional AutoEncode (CAE) using Visible Light Communication (VLC). To overcome the issue of fluctuated signal reading in the RSS-based fingerprint method, the authors have proposed an algorithm taking into account a set of consecutive signal readings and converting them to an RSS Temporal Image (RTI), instead of implementing the traditional RSS measurement processing technique. However, despite having been used in several works [31,32], VLC suffers from issues such as interference with other ambient lights, signal shadowing and usually requires the receiver to be in Line-Of-Sight (LOS), which can affect the accuracy of the location estimation. A detailed comparison of deep learning and other machine learning algorithms used in localization for IoT environment is covered in [33,34].

Until now, most of the existing IoT-based indoor localization solutions have mainly focused on either 2D localization or floor detection. However, in some special use cases scenarios such as indoor navigation for Unmanned Aerial Vehicle (UAV) or Automated Guided Vehicle (AGV) in a smart factory or big supermarket, precise 3D positioning is indispensable for daily operations. To address this issue, we suggest the DELTA to maximize the localization accuracy and minimize the distance error in a 3D indoor environment.

## 3. System Model and 3D Localization Problem

In this section, we introduce our proposed system model using Deep Neural Networks (DNN) and multi-layered radiomap to perform 3D Indoor Localization. To the best of our knowledge, this is a novel approach to implement deep learning on multi-layered radiomap for localization purposes. The main benefit of the proposed method is improved localization accuracy, and computational complexity minimization during online fingerprinting through the adoption of deep learning techniques, while at the same time utilizing the widely spreading WSN and/or IoT infrastructure making it an economical solution. To realize these steps, we considered N to be the number of transmitters in the environment and x, y and z, the corresponding coordinates of each fingerprint entry on the constructed radiomap. The 3D multi-layered fingerprint database has been constructed by linking the RSS values received from the transmitters to a 3D location on the radiomap [35]. This can be mathematically expressed as:

(1) $$M=\{\left(L_{1},S_{1}\right),\left(L_{2},S_{2}\right),\ldots ,\left(L_{n-1},S_{n-1}\right),\left(L_{n},S_{n}\right)\}$$

where *M* is the ratio-map database, $S\in R^{NXM}$ is a vector of RSS signal values and L is a vector of three values: L≡{x,y,z} and $L_{n}$ represents the total number of the sample location of $x_{n}$, $y_{n}$ and $z_{n}$ associated with each signal vector sample $S_{n}$ collected during the offline-phase.

In this respect, the estimation problem is defined by solving the 3D localization problem using a matrix of historical location points and their corresponding signal values. However, the challenge is to model the non-arbitrary relationships between *N* transmitters members of *S* signal matrix to predict accurately the 3D location L using a deep learning algorithm. To achieve this, the 3D localization has been segmented to two sets of problems:

**Problem** **1.** *Given a matrix of S signal sent from N transmitters, predict the x and y coordinates of a 2D mobile station location. This can be written as:*

(2) $$\lambda_{1}=f\left({\bar{S}}_{ij}\right)$$

*where $\lambda_{1}$ represents the $x_{i}$ and $y_{i}$ 2D location, which we would like to estimate, and $f(S_{ij})$ represents the function that utilizes RSS values received by the transmitters to predict the location of the mobile station.*

**Problem** **2.** *Given a matrix of S signal sent from N transmitters to the mobile station and $x_{i}$, $y_{i}$, known from problem 1, estimate the $z_{i}$ coordinate. This can be mathematically expressed as:*

(3) $$\lambda_{2}=f\left({\bar{S}}_{ij},\lambda_{1}\right)$$

*where $\lambda_{2}$ is the $z_{i}$ location, $\lambda_{1}$ is the output of problem 1 solution and $S_{ij}$ represents a matrix of signal values S as previously stated in problem 1.*

## 4. DELTA 3D Localization for 5G WSN Network

**Figure 1:**
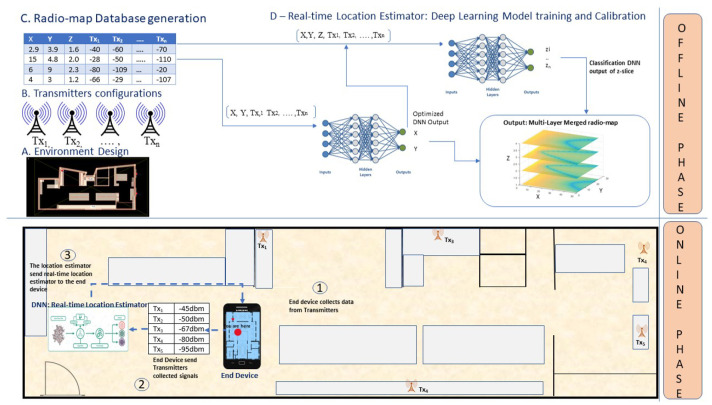
Detailed architecture of deep learning-based co-operative architecture (DELTA).

In this section, the DELTA System has been developed for 3D multi-layered indoor environment localization. Figure 1 depicts the steps undertaken to realize a co-operative system for accurate 3D prediction.

### 4.1. Test Environment Description

In this subsection, we describe the test environment. The area of interest is a typical laboratory, with open spaces as well as private rooms defined by the following dimensions: 8 m width × 16 m depth × 2.75 m height. The lab environment was dynamic during this experiment.

#### 4.1.1. Step I: The Physical Network Setup

**Figure 2:**
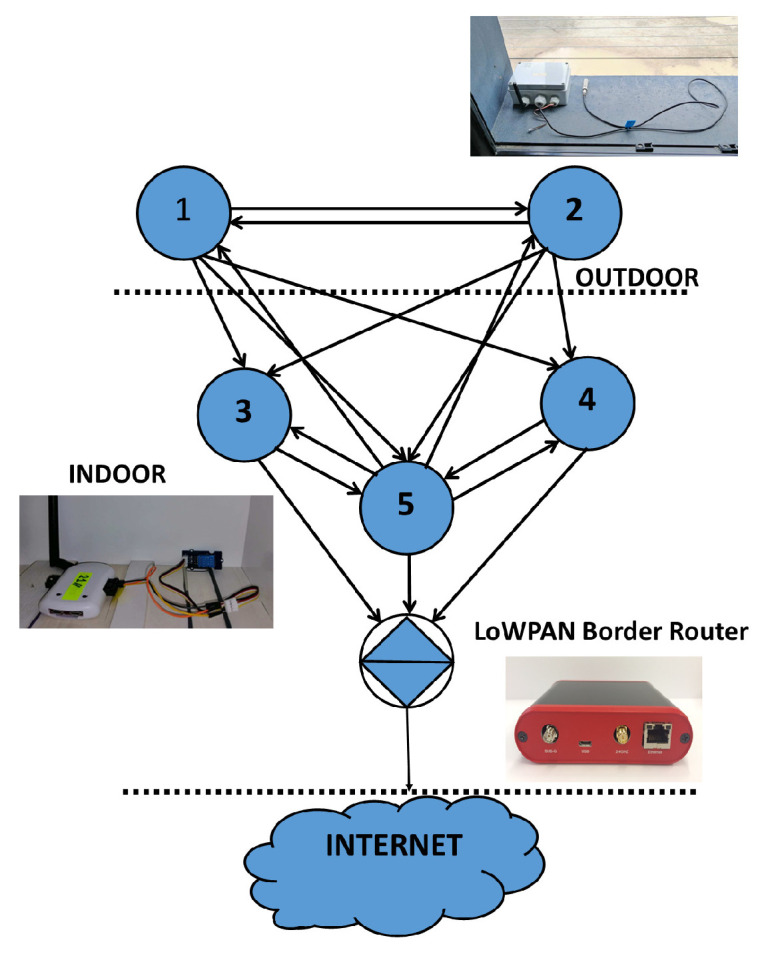
Network setup topology.

For the physical setup, an indoor test environment was deployed where a 5G network was emulated by a typical IoT network with Zolertia RE-Mote Revision B nodes connected to a LoWPAN Border Router, as illustrated in Figure 2.

We randomly placed 5 Zolertia nodes, with their antennas at vertical polarization, as shown in Figure 2. The nodes and the ray tracing propagation mechanisms have been configured as per Table 1 and Table 2.

#### 4.1.2. Step II: Connecting IoT to 5G C-RAN

**Figure 3:**
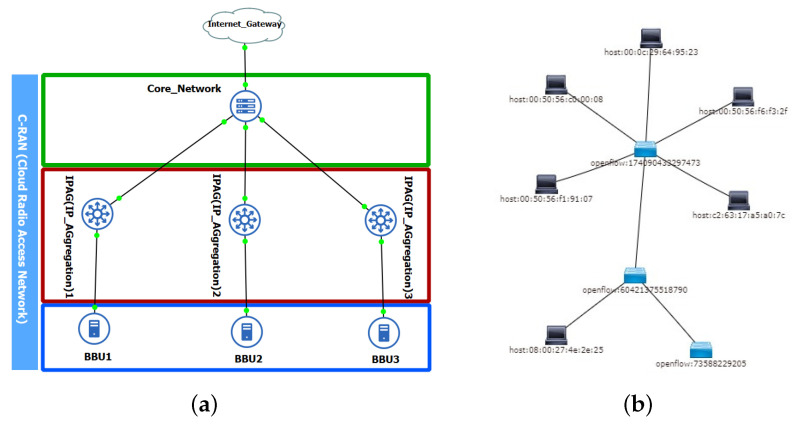
5G C-RAN setup on GNS3 and WSN Network Connected to OpenDaylight SDN Controller. (**a**) 5G emulated C-RAN testbed on GNS3; (**b**) WSN and GNS3 emulated 5G C-RAN connected on OpenDaylight.

To simulate the 5G WSN environment, each Zolertia node was connected to an experimental 5G C-RAN. The setup was built using a GNS3 network simulator [36] and OpenDaylight Software Defined Controller [37]. These two can control the network setup behaviour at the network layer level. Figure 3 shows a setup built using a GNS3 network simulator and a Software Defined Controller OpenDaylight dashboard for the network topology. These two elements can control the network setup behaviour at the network layer level.

#### 4.1.3. Step III: Simulating the Test Environment

**Figure 4:**
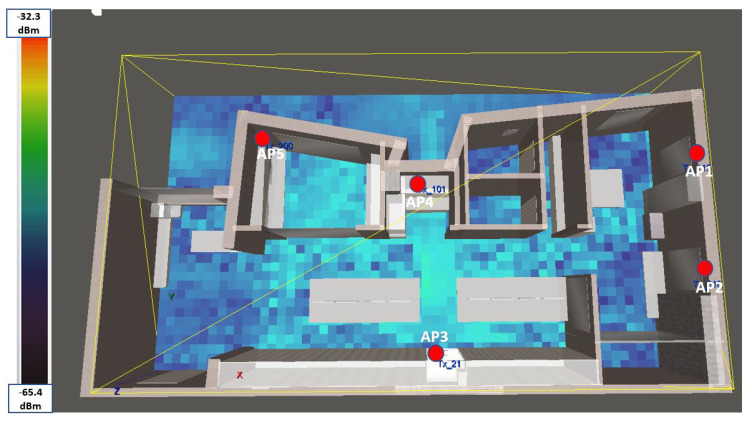
TruNET Wireless Simulator Radiomap for Access Point 3.

Using a 3D deterministic simulator called TruNET Wireless [38], we constructed a multi-layered fingerprint radiomap dataset, in order to conduct the offline training phase, as illustrated in Figure 1. During this procedure, in addition to the network setup configuration, the constitutive parameters of all environment object materials were also configured as per Table 2, in order to retrieve realistic results [39]. The benefits of utilizing a deterministic simulation are to construct radiomaps instead of launching measurement campaigns as analysed in [40]. The summary of correlation results of this study is covered in Section 5. The simulation environment for this study is shown in Figure 4.

#### 4.1.4. Physical Network Behaviour

**Figure 5:**
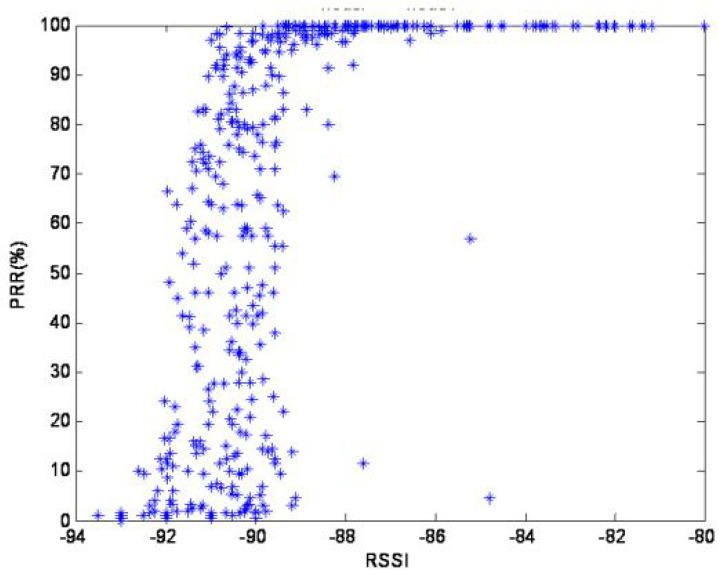
Packet Reception Ratio (PRR) vs. Received Signal Strength Indicator (RSSI) Signal CC2420 chip Baccour et al. [41].

The signal propagation can be affected by various factors leading to the degradation of the signal quality especially in low power radio networks such as Wireless Sensor Networks. For a successful simulation, it is always crucial to observe the physical network behaviour during the offline measurement campaign. The effects of the physical layer and the various factors contributing to changes in the environment have been extensively studied in [41]. Using Link Quality Estimation (LQE) metrics such as Packet Reception Ratio (PRR) and Signal-to-Noise-Ratio (SNR), Baccour et al. [41] have studied the factors affecting a transmitter chip similar to that used in this experiment. It is very crucial to note that the simulated environment can be affected by various changes happening at the physical network. For the nodes used in this simulation, Figure 5 shows how the change in the Received Signal Strength Indicator (RSSI) can affect the PRR.

**Figure 6:**
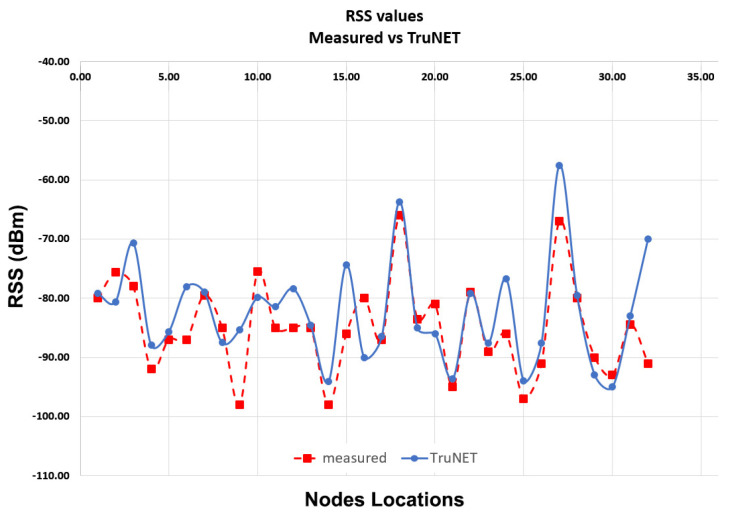
RSS Values Measured vs. TruNET Kanaris et al. [42].

Sometimes, measurement campaigns can be affected by various environmental noises, which may lead to unrealistic readings, either due to signal spikes or fluctuations. This noise can be either thermal noise or interference from other people’s equipment operating at the same frequency. To ensure signal samples obtained from TruNET Wireless are realistic, Figure 6 depicts the RSS coverage correlation analysis experiment conducted in [42]. The samples have been collected from Zolertia nodes over a week period at different instances with an interval of 15 min for each sample. The produced measurements for each RP have been averaged using the mean value. During this experiment, the IoT nodes have always been fixed and the environment was dynamic with people moving around.

Finally, it is clearly indicated that the simulated RSS values from the TruNET wireless simulator highly approximates the measured ones reaching a correlation level of more than 73%.

### 4.2. DELTA Architecture

Deep learning is a fundamental building block of the proposed architecture. It allows computational models consisting of multiple processing layers to learn the representation of data within multiple abstract levels [43]. One of the most important elements of deep learning is deep neural networks. Bengio et al. [44] refer to this as either deep feed forward networks or Multiple Layers Perceptron (MLP) since they have more than two hidden layers.

**Figure 7:**
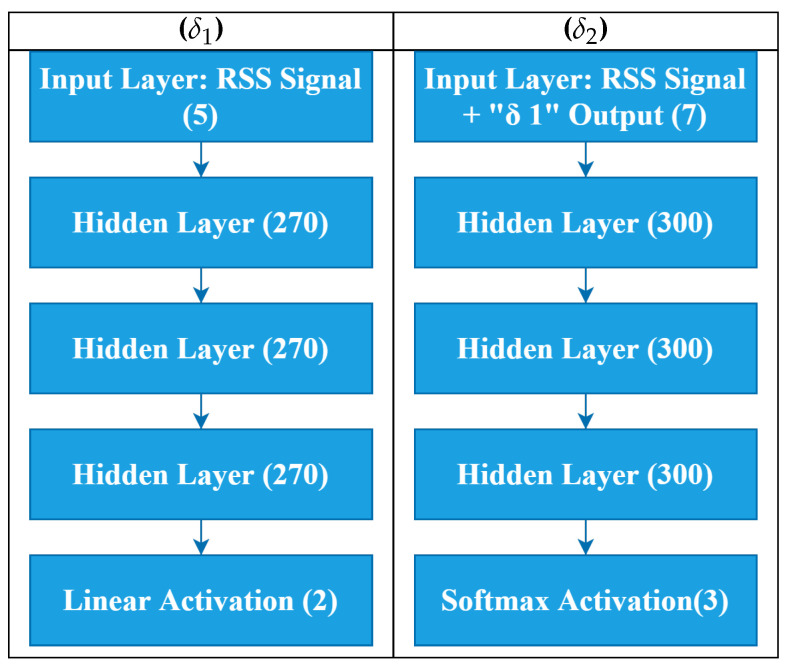
Layers of DELTA Architecture Network.

Our proposed architecture, as illustrated in Figure 7, consists of two deep neural networks. The first is a regression model $\delta_{1}$ used to predict the 2D location of a mobile device. The second is a classification model referred to as $\delta_{2}$. Figure 7 illustrates the number of layers, neuron, input and output parameters used for both models. Based on numerous trials and hyper-parameters tuning, we observed that three hidden layers were the best fit model for both networks.

### 4.3. DELTA Layers

#### 4.3.1. Input Layers

For $\delta_{1}$, the input is a transposed vector of RSS signals that can be expressed as follows: $S={\left[s_{1},s_{2},\ldots ,s_{n}\right]}^{T}$. For $\delta_{2}$, the input is slightly different to $\delta_{1}$. It consists of RSS signal input *S* and the output of $\delta_{1}$. Each observation has a set of signals and predicted locations. This can be written as:

(4) $$\delta_{2\_input}=S\cup \left(L_{x},L_{y}\right)$$

where S is the signal and $L_{x},L_{y}$ are the corresponding x and y locations. These two values are approximated using $\delta_{1}$, as shown in Figure 7.

#### 4.3.2. Hidden Layers

Each element of this input gets multiplied by its specific weight vector $\vec{w}$ and the product is added to a bias *b*. For the first hidden layer, this is expressed as follows:

(5) $$h1=\sum_{i=1}^{n}w_{i}^{1}I_{i}+b_{i}^{1}$$

where $I_{i}$ is an element from the input vector. Each $I_{i}$ represents an input from a transmitter in the constructed fingerprint database. A summation of all these inputs is then fed to an activation unit A. In this case, the type of activation function used is a called Rectified Linear Unit (ReLu).

(6) $$A_{1}=max\left(0,h1\right)$$

where $A_{1}$ is an activation unit for the first hidden layer. The output of this hidden layer is the number of hidden neurons specified in the first hidden layer. Similarly, Equation (7) for hidden layer 2 is expressed as follows:

(7) $$h2=\sum_{i=1}^{n}w_{i}^{2}a_{i}^{1}+b_{i}^{2}$$

This result is then fed into a further activation unit $A_{2}$:

(8) $$A_{2}=max\left(0,h2\right)$$

The hidden layer three receives the output of Equation (8) and makes similar calculations to h2:

(9) $$h3=\sum_{i=1}^{n}w_{i}^{3}a_{i}^{2}+b_{i}^{3}$$

Finally, the results returned in Equation (9) are fed into the activation unit $A_{3}$.

(10) $$A_{3}=max\left(0,h3\right)$$

#### 4.3.3. Output Layers

For $\delta_{1}$ model, since the desired output is a real-valued number, a linear function has been applied using the following equation:

(11) $$g\left(y=j|a_{i}\right)=\sum_{i=1}^{n}w_{i}^{4}a_{i}^{3}+\epsilon_{i}$$

For $\delta_{2}$ model, the output is multiple class labels, therefore the Softmax function equation below has been used:

(12) $$\theta \left(a_{i}\right)=\frac{\exp(a_{i}^{3})}{\sum_{j}\exp\left(a_{j}^{3}\right)}$$

To get the best final approximation, $\delta_{1}$ supports $\delta_{2}$. Algorithm 1 explains how both networks cooperate to make a final localization.
**Algorithm 1:** DELTA Algorithm for 3D Localization
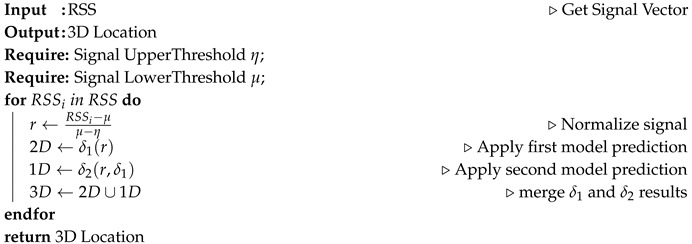


### 4.4. Prepossessing

#### 4.4.1. Fingerprints Radiomap Database

As previously mentioned, we began by constructing the radiomap database using eight features. Table 3 gives a detailed explanation of each variable.

**Figure 8:**
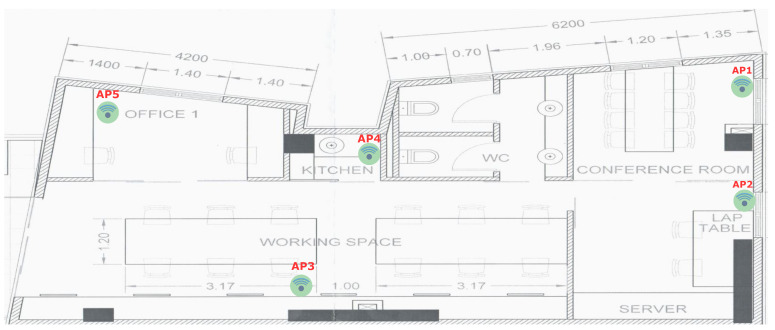
Access points position on the setup environment floor-plan.

The constructed radiomap consists of 2880 3D References Points (RPs) associated with RSS values from five different WSN Access Points(APs). Each AP is placed at least three meters away. The position of these APs is shown on the lab floor-plan illustrated in Figure 8.

**Figure 9:**
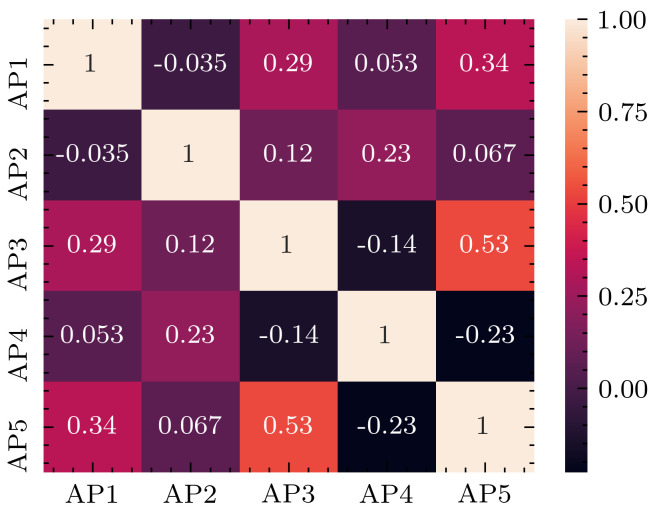
WSN access points correlation matrix.

To ensure that there is no redundancy in the information collected, a Pearson correlation test has been conducted between each AP and the result is shown in Figure 9. There is clearly no high negative or positive correlation between the APs used in this experiment.

**Figure 10:**
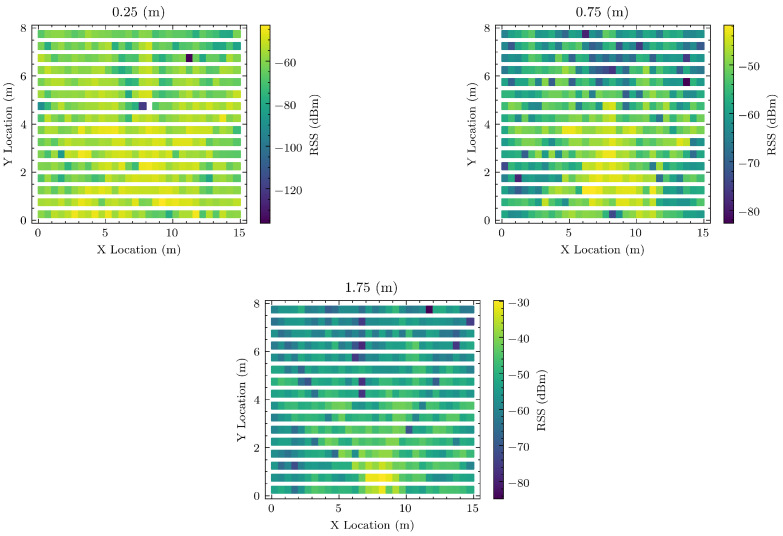
Signal strength map for WSN access point 1 for each Z layer.

In addition to this, Figure 10 shows each layer on the radiomap database constructed is significantly different from the other layer. The Figure shows the signal at 0.25, 0.75 and 1.75 m for Access Point 1.

#### 4.4.2. One-hot Encoding

One-hot encoding is one of the most common techniques for converting a token into a vector [45]. The conversion is achieved by associating each unique integer with every unique value from the column z. This turns every unique value into a binary vector having the size of the unique values. As a result, every column will have zero except for where the unique value has occurred. In our case, we have used the steps followed in Algorithm 2 to one-hot encode our target variable:
**Algorithm 2:** One hot Encoding
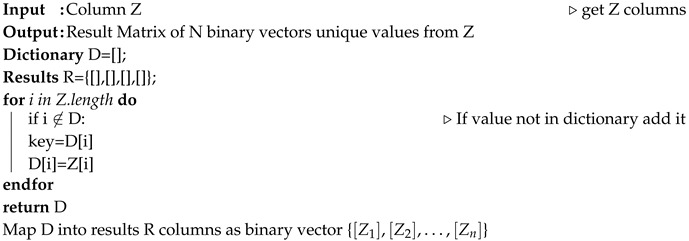


#### 4.4.3. Min–Max Normalization

Min–Max normalization has been implemented to make sure the learning of signal representation data is faster for DELTA architecture models to converge quickly. This concept works by fitting the original data into a new scale between 0 and 1. After this numeric transformation, the highest value becomes close to 1 and the lowest value is close to 0 as stated in [46]. The formula used to achieve this, is the following:

(13) $$\frac{RSS_{i}-min\left(RSS\right)}{min(RSS)-max(RSS)}$$

where min(RSS) represents the values minimum threshold signal specified during the training signal, i.e., −120 dBm and max(RSS) represents the maximum value measured, i.e., −30 dBm. Each signal measurement we want to convert is denoted by $RSS_{i}$ where *i* is the $i^{th}$ row in N Transmitter. For other scenarios, it is advisable to use the receiver sensitivity level as the minimum value and the strongest measured signal during the offline-phase as the maximum value.

### 4.5. Hyper-Parameters Fine-Tuning

#### 4.5.1. Loss Functions

- Using Euclidean Distance as loss function for $\delta_{1}$ model, the purpose is to train the model to minimize the Mean Euclidean Distance (MED) error between the actual and the predicted location.

(14) $$D\left(L^{act},L^{pred}\right)=\frac{1}{M}\sum_{n=1}^{m}\sqrt{{\left(x_{j}^{act}-x_{j}^{pred}\right)}^{2}+{\left(y_{j}^{act}-y_{j}^{pred}\right)}^{2}}$$

$L^{act}$ here denotes the actual location and $L^{pred}$ denotes the predicted location.

- For the $\delta_{2}$ model, Categorical Cross-entropy is implemented as a loss function. This can be written as:

(15) $$H\left(L^{act},L^{pred}\right)=-\sum_{j=0}^{M}\sum_{i=0}^{N}(z_{ij}^{Act}\cdot log\left(z_{ij}^{pred}\right)$$

where $L^{act}$ denotes the actual location and $L^{pred}$ denotes the predicted location. While $z_{ij}$ denotes the ith observation in the jth z output class or level.

#### 4.5.2. Hidden Layers and Neurons Size Determination

**Figure 11:**
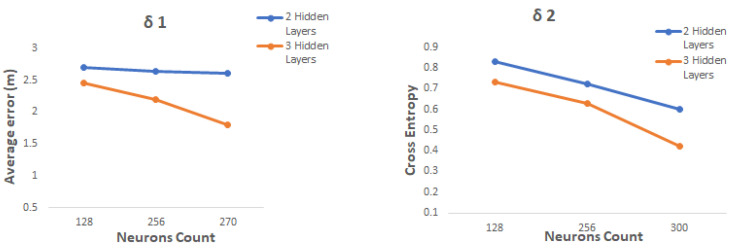
The number of hidden layers and neurons vs. each loss function.

The number of hidden layers and neurons count used in the DELTA has been determined using the loss function specified the previous subsection. Figure 11 shows the performance of each network for each neuron count and layers number selected. As demonstrated in this figure, the categorical cross entropy loss is minimized after a third hidden layer has been added and the neurons count has been set to 300. Similarly, the average error was decreased in delta one after the parameters were changed to 300 neurons and three hidden layers.

#### 4.5.3. Batch Normalization

A batch is the number of samples propagated through the neural network model before the parameters are updated. To train each neural network faster, we have supported each layer with a batch normalization. This sort of normalization is applied to input samples of the same batch size. This fine-tuning technique has been proven to speed up the training and learning process by 12 times faster than the normal architecture as described by authors in [47]. The formula for the batch normalization implemented on each Deep Neural Network of DELTA system is:

(16) $$T_{i}=\frac{(T_{i}-\mu \left(T\right)}{\sqrt{\sigma^{2}\left(T\right)+\epsilon }}$$

where *T* is training batch, μ(T) is its mean, $\sigma^{2}\left(T\right)$ is its variance and ϵ is a small constant number added to support the variance. For this to work in Keras deep learning library [45], a layer of batch normalization with explicit parameters has to be added at the beginning of each hidden layer.

#### 4.5.4. Regularization

To avoid overfitting, a regularization technique has been implemented to switch off certain neurons for some layers. This technique is called dropout. Details for this technique are provided by Nitish et al. in [48]. The dropout rate used in DELTA is 0.20 as suggested by [48]. After experimentation, we have concluded that for better results are achieved when implementing batch normalization before dropout.

### 4.6. Optimization

Optimization is the process of training a network using mini-batches and iterations to get the optimum configuration for its parameter. One of the widely used stochastic optimization algorithms in deep learning is ADAptive Momentum (ADAM). The algorithm can be viewed as a combination of RMSprop and Momentum [49]. It works by correcting the bias b and the weight w after each iteration. To get the best results from ADAM’s parameters, we specified a learning rate α=0.001, $\beta_{1}$ = 0.9 for the momentum control, $\beta_{2}$ = 0.99 for squared weight in RMSprop section and $\epsilon =10^{-8}$ as specified by the authors in [49]. To implement this in Keras, ADAM parameters have to be specified before the model is compiled.

### 4.7. Scoring

**Figure 12:**
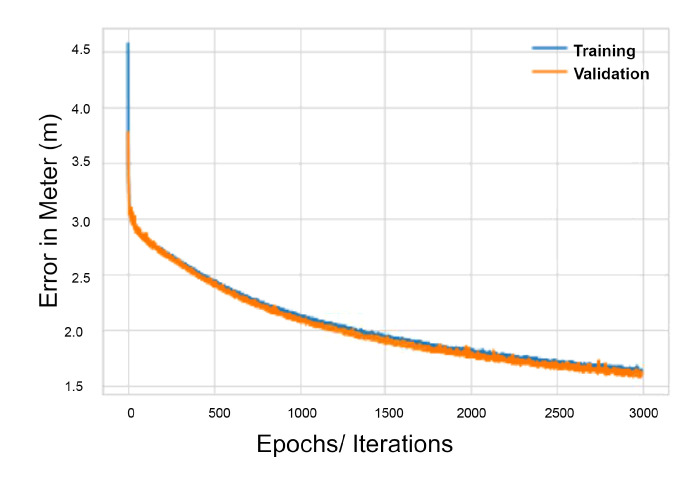
$\delta_{1}$ Model mean Euclidean distance error in meter (m) vs. the number of Epochs.

Using 900 hidden neurons and three hidden layers, we have constructed model $\delta_{1}$ to predict x and y locations. This has yielded 279,302 parameters to be trained. Our cost function is the euclidean distance difference between each predicted observation and the original location. To minimize it, hyper-parameters have been fine-tuned such as the batch sizes and the number of times an algorithm will iterate through an entire training dataset. One iteration is referred to as epochs. The aforementioned methodology resulted in an average positioning error of 1.6 m average (less than 2 m error overall) in both training and validation phases. Figure 12 shows how the $\delta_{1}$ model mean Euclidean distance error in meters decreases over the number of epochs chosen, in this case 3000 epochs. However, by the end of epoch 3000, the model has converged and stopped improving its accuracy.

**Figure 13:**
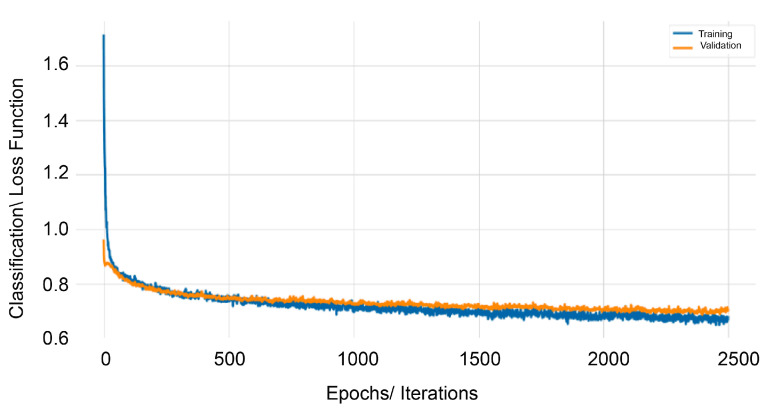
$\delta_{2}$ Categorical cross-entropy vs. the number of Epochs.

Similarly, after an iterative tweaking of the architecture parameters, using 810 number of neurons and three hidden layers, we have constructed model delta 2 where z layer is the target variable. A total number of 235,592 parameters were trained in this model. The cost function is the multi-categorical cross entropy, which is used widely for classification scoring. Figure 13 shows how the categorical cross-entropy has been minimized after 2500 epochs.

## 5. Performance Evaluation Results

In this section, we explore, evaluate and critically analyse the simulation results against famous industry methods such as SVM and KNN. However, before going through the results analysis, it is worth mentioning that KNN and SVM modelling tasks have been carried out using Scikit-learn [50], a widely used Python library toolset for machine learning and statistics. More specifically, SVM models have developed using an SVM class from the Scikit-learn library and KNN models have been built using a classifier class called KNeighborsClassifier [51]. The DELTA models have been constructed using Keras API [52], a deep learning library also available in Python. During the evaluation phase, the three algorithms were implemented using python software on the same machine with Intel i7-4790@3.60GHz CPU and 16 GB of RAM. In terms of time complexity, KNN has finished after 230 ms while SVM has taken 450 ms. The proposed DNN has used 160 ms to execute, making it more efficient than KNN and SVM.

### 5.1. Results Analysis

#### 5.1.1. $\delta_{1}$s. KNN and SVM

**Figure 14:**
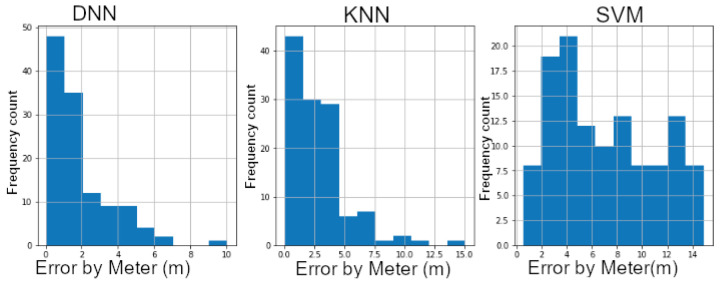
$\delta_{1}$ vs. K-Nearest Neighbor (KNN) vs. Support Vector Regression (SVR).

Using 180 random samples [39], we have bench-marked and assessed DNN model $\delta_{1}$ against KNN and Support Vector Regression (SVR) models. The samples have been obtained for each z layer making a total of 540 RPs. The SVR has been trained using a linear kernel, a degree of one and an epsilon value of one using 80% training and 20% validation data sets. Similarly, a KNN model has been trained with a K value set to three. The results in Figure 14 show the error distribution in meters for all three models. SVR has scored a rather worse error distribution where the peak of its distribution ranges between 4 and 6 m error. KNN has done slightly better compared to SVR. However, a large proportion of the distribution error falls between 3 and 5 m, which makes it the second worse performing after SVR. DNN $\delta_{1}$ has performed better. The peak of its distribution error samples falls between zero and two meters with a mean error of 1.6 m. A detailed result is provided in Table 4.

#### 5.1.2. $\delta_{2}$s. KNN and SVM

**Figure 15:**
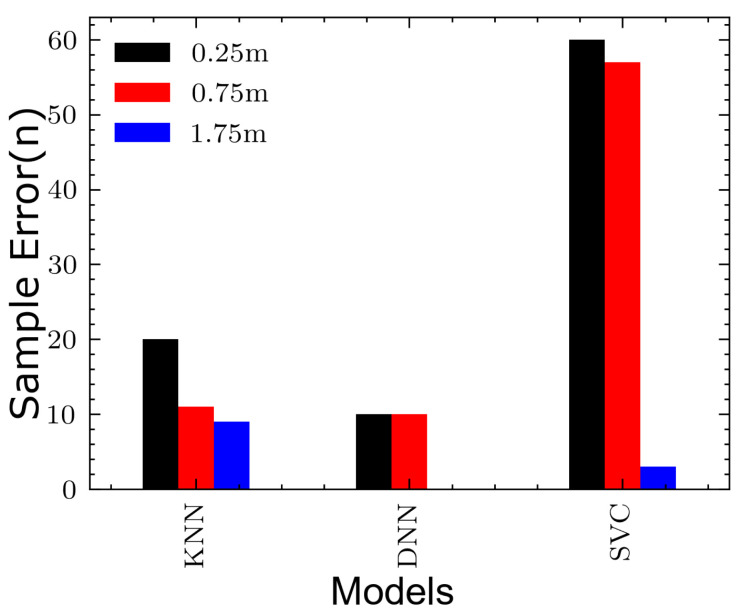
Model comparison: $\delta_{2}$ vs. KNN vs. SVC.

Using the aforementioned samples, the z layer (z coordinate) has been estimated. The results are depicted in Figure 15 illustrating a visual comparison of each classifier in a bar-chart using misclassification count as a measure. Each model has been given an equal number of three classes 0.25, 1.25 and 1.75 m. At first glance, Figure 15 shows that Support Vector Classifier (SVC) has performed very badly in terms of classification of observations. The model has failed to accurately classify during the online phase. More than 66%—circa 120 samples—have been wrongly classified. With a total of 40 misclassified samples, K-Nearest Neighbor (KNN) has performed better than SVC but still does not differentiate between certain classes properly. Our proposed $\delta_{2}$ model of DNN, has made excellent classification compared to both later models. As an effect, 100% of the 0.25 m layer has been accurately classified while more than 95% of the other two classes, 1.25 and 1.75 m, have also been properly predicted. The total number of misclassified samples is 20 bringing the classification accuracy rate to 89%. This shows how the proposed 3-D multi-layered model has outperformed the traditional models.

Table 5 gives a detailed count of each model and its misclassification count. The worse performing model is highlighted in red and the best performing model is highlighted in blue.

## 6. Conclusions

In this work, we have proposed a novel approach for 3D Indoor Localization using DNN cooperative network algorithms implemented on 3D multi-layer radiomaps. To emulate a 5G infrastructure IoT indoor scenario, an IoT network is interconnected to an experimental 5G C-RAN. Using only an offline fingerprint database, we have also demonstrated how the proposed model has outperformed traditional industry models such as KNN. We have accurately implemented this model to the indoor environment. If the steps shown in Figure 1 and Figure 7 are properly followed, a reliable and fast 3D localization can be achieved. This concept can also be further developed to cover more complex indoor positioning scenarios, involving radio data from a heterogeneous network (HetNEt) such as 5G microinfrastructure (Microcells, femtocell, picocells, etc.). Finally, the proposed DELTA model works very well with RSS based IoT and Wireless Sensor Networks. Thus, our future work will be improving the model by including information fused from other networks such as WiFi and Bluetooth Low Energy (BLE) and experimenting with more vertical layers. Another research direction could be adding floor level detection for buildings with multiple floors.

## Figures and Tables

**Table 1:** Wireless Sensor Network (WSN) and radio propagation parameters.

Parameter	Value
**Rx sensitivity (dBm)**	−120
**Tx power (dBm)**	3
**antenna Type**	omni
**Max refractions**	12
**Max reflections**	12
**Max diffractions**	1

**Table 2:** Material constitutive parameters of the test environment.

Material	El. Per. (F/m)	L. Tangent
**Concrete**	3.9	0.23
**Wood**	2	0.025
**Brick**	5.5	0.03
**Metal**	1	1,000,000
**Plasterboard**	3	0.067
**Glass**	4.5	0.007

**Table 3:** The features used to construct the fingerprints database.

Variable	Min. Value	Max. Value	Type
**X**	0	8	coordinates
**Y**	0	16	coordinates
**Z**	0.25	1.75	coordinates
**AP1**	−120 dBm	−28 dBm	RSS value
**AP2**	−100 dBm	−30 dBm	RSS value
**AP3**	−100 dBm	−40 dBm	RSS value
**AP4**	−90 dBm	−50 dBm	RSS value
**AP5**	−100 dBm	−60 dBm	RSS value

**Table 4:** Frequency Count of Distance error (m) for each model.

	DNN	KNN	SVM
**Less Than 2 m**	79	51	9
**Between 2 m and 7 m**	39	64	60
**More than 7 m**	2	5	51

**Table 5:** Misclassification count for each model.

Model	0.25 m	0.75 m	1.75 m
**DNN**	10	10	0
**KNN**	20	11	9
**SVC**	60	57	3

## Abbreviations

The following abbreviations are used in this manuscript:

3/2D	3/2 Dimensions
5G	5th Generation
AoA	Angle of Arrival
ADAM	ADAptive Momentum
AP	Access Points
BLE	Bluetooth Low Energy
C-RAN	Cloud-Radio Access Network
CAE	Convolutional AutoEnconde
CD-1	Contrastive Divergence with one-step iteration
CNN	Convolutional Neural Networks
D2D	device-to-device
DBN	Deep Belief Network
DELTA	DEep Learning cooperaTive Architecture
DNN	Deep Neural Network
DoA	Direction of arrival
GNSS	Global Navigation Satellite System
GPS	Global Positioning System
HetNet	Heterogeneous Network
IoT	Internet of Things
KNN	K-Nearest Neighbor
LOS	Line-Of-Sight
MED	Mean Euclidean Distance
MDPI	Multidisciplinary Digital Publishing Institute
MLP	Multiple Layers Perceptron
mm-Wave	Millimeter Wave
PPDR	Public Protection and Disaster Recovery
PRR	Packet Reception Ratio
RP	Reference Point
RSS	Received Signal Strength
RSSI	Received Signal Strength Indicator
RTI	RSS Temporal Image
RTOF	Return Time of Flight
SNR	Signal-to-Noise-Ratio
SVC	Support Vector Classification
SVM	Support Vector Machine
SVR	Support Vector Regression
TDOA	Time Difference of Arrival
TOA	Time of Arrival
ToF	Time of Flight
V2X	Vehicle-to-Everything
VLC	Visible Light communication
WSN	Wireless Sensors Networks
